# The burden of subclinical TB in Nigeria

**DOI:** 10.5588/pha.24.0038

**Published:** 2024-12-01

**Authors:** B. Odume, C. Ogbudebe, Y. Mukadi, C. Dim, E. Chukwu, O. Chukwuogo, S. Useni, N. Nwokoye, M. Sheshi, D. Nongo, R. Eneogu, A. Ihesie, E. Ubochioma, C. Anyaike

**Affiliations:** ^1^ KNCV Tuberculosis Foundation, Abuja, Nigeria;; ^2^ United States Agency for International Development (USAID), Washington, DC, USA;; ^3^ College of Medicine, University of Nigeria, Ituku-Ozalla, Nigeria;; ^4^ USAID, Abuja, Nigeria;; ^5^ National TB, Leprosy and Buruli Ulcer Control Programme, Federal Ministry of Health Nigeria, Abuja, Nigeria.

**Keywords:** latent tuberculosis, active tuberculosis, digital X-ray with artificial intelligence, asymptomatic presumptive

## Abstract

**SETTING:**

This study is a retrospective review of a large-scale systematic TB screening project conducted in six states of Nigeria.

**OBJECTIVE:**

To determine the magnitude and characteristics of subclinical TB and the relative contributions of bacteriological versus clinical diagnosis in its identification in Nigeria.

**DESIGN:**

Data were retrospectively analysed from six states of Nigeria, where parallel screening with any TB symptoms and chest X-ray (CXR) with artificial intelligence (AI) was used for active case finding. Diagnosis of TB among presumptive was confirmed using either bacteriological tests or clinical review of CXR.

**RESULTS:**

Out of 8,516 presumptive identified during the project, 172 (2.0%) had no TB symptoms (males: 73.8%, females: 26.2%). The overall prevalence of TB among all presumptive was 21.9% (*n* = 1,867), including 62 (3.3%) subclinical TB and 1,805 (97.3%) active TB cases. The proportion of clinical diagnosis using CXR was significantly higher in the subclinical TB group than in the active TB group (79.0% vs. 63.5%; *P* = 0.012, OR = 2.2, 95% CI 1.17–4.03).

**CONCLUSION:**

Subclinical TB contributed 3.3% of the large TB burden in this study (22 per 100 presumptive). These cases would have been missed if only symptom-based TB screening had been employed.

## Abstract (fr)

**CONTEXTE:**

Cette recherche constitue une analyse rétrospective d'un programme de dépistage systématique de la TB à grande échelle réalisé dans six États du Nigeria.

**OBJECTIF:**

Évaluer l'étendue et les particularités de la TB subclinique ainsi que les apports respectifs du diagnostic bactériologique par rapport au diagnostic clinique dans son identification au Nigeria.

**MÉTHODES:**

Les données ont été examinées de manière rétrospective dans six États du Nigéria, où un dépistage simultané de tout symptôme de TB et de la radiographie pulmonaire (CXR) assisté par intelligence artificielle (AI) a été mis en œuvre pour la recherche active de cas. Le diagnostic de TB chez les patients présumés a été validé par des tests bactériologiques ou par une évaluation clinique de la CXR.

**RÉSULTATS:**

Parmi les 8 516 individus présumés identifiés durant le projet, 172 (soit 2,0%) ne manifestaient aucun symptôme de TB (73,8% d'hommes et 26,2% de femmes). La prévalence totale de la TB parmi l'ensemble des cas présomptifs s'élevait à 21,9% (*n* = 1 867), comprenant 62 (3,3%) cas de TB subclinique et 1 805 (97,3%) cas de TB active. La proportion de diagnostics cliniques par CXR était significativement plus élevée dans le groupe des cas de TB subclinique par rapport à celui des cas de TB active (79,0% contre 63,5% ; *P* = 0,012, OR = 2,2 ; IC à 95% 1,17–4,03).

**CONCLUSION:**

La TB subclinique a représenté 3,3% de la charge significative de morbidité associée à la TB dans cette étude (22 pour 100 cas supposés). Ces cas seraient restés non identifiés si le dépistage de la TB s'était uniquement fondé sur les symptômes.

Pulmonary TB is historically categorised into latent or active infection states that were believed to be asymptomatic and non-infectious versus symptomatic and infectious, respectively.^1^ This traditional binary classification failed to recognise that there are active bacteria in both the latent and active states such that a proportion of individuals with no recognisable TB symptoms can transmit the infectious agent,^2^ and thus would be missed during symptom-only TB screening. The four primary TB symptoms are cough, fever, night sweats, or weight loss; using the presence of any one of the symptoms for TB screening has an estimated sensitivity and specificity of respectively 71% and 64%.^3^ Thus, a large proportion of all TB-diagnosed persons (bacteriologically and/or radiographically) are negative for TB symptoms screening.

Consequently, there is a new concept for TB disease progression spectrum starting from latent TB through incipient, subclinical TB (no symptom or symptom not recognised) to active TB with recognisable symptoms (Figure 1).^1,4^ Subclinical TB has no recognisable TB-related symptoms but causes other abnormalities that can be detected using existing radiologic or microbiologic tests.^4^

**FIGURE 1.**
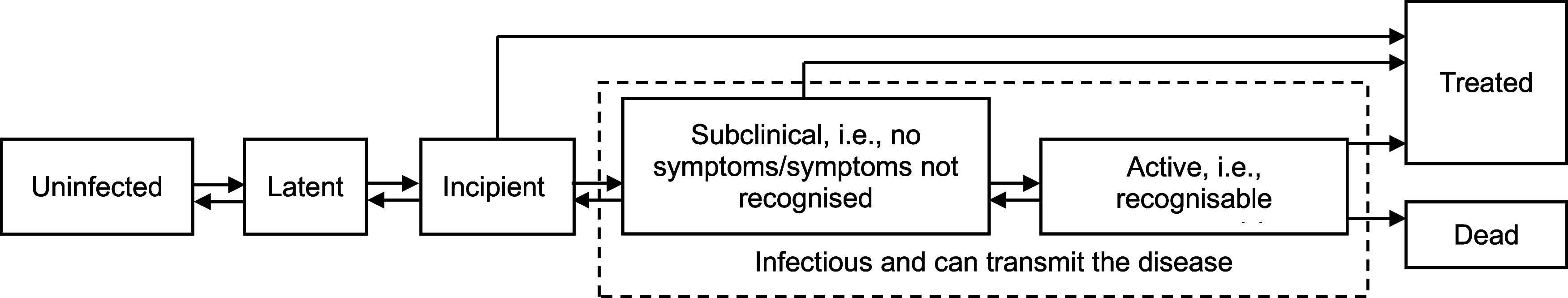
Current TB disease spectrum concept.^1^

As illustrated in Figure 1, subclinical TB can resolve into incipient TB that is not infectious, persist as subclinical TB, which is infectious, or progress to overt symptomatic (active) TB that is also infectious. Unfortunately, most active TB case-finding (ACF) interventions employ symptom-only TB screening methods, thereby leaving out the large pool of infectious subclinical TB with obvious implications.

The burden of subclinical TB appears to be enormous, with an estimated global prevalence of at least 10 million; thus, it may be responsible for a large proportion of ongoing TB transmission in the communities.^1^ A hospital-based report from Suzhou, China, showed a sub-clinical TB percentage of all TB cases of 18.2%,^5^ while a systematic review of 23 national surveys and five subnational surveys from 23 countries in Africa and Asia found a percentage of subclinical TB range of 36.1 to 79.7% (median = 50.4%).^6^ Also, subclinical TB can occur as recurrent TB; in South Africa, the TRuTH study found a subclinical TB rate of 4.2% among adult TB-HIV-coinfected patients who had completed their TB treatment after a median follow-up of 41 months.^7^

Nigeria’s National Tuberculosis and Leprosy Control Programme (NTLCP) guidelines categorise TB using the traditional binary TB classification (i.e., latent and active).^8^ Although the guidelines promote the use of chest X-ray (CXR) for TB screening of adults during community-based ACF interventions, TB screening in Nigeria was routinely symptom-based before the introduction of portable digital X-ray (PDX) with artificial intelligence (AI) by KNCV Tuberculosis Foundation Nigeria (KNCV Nigeria) for systematic TB screening in Nigeria.^9.10^ Because a substantial proportion of TB transmission in the communities is speculated to originate from individuals with subclinical TB, the end TB epidemic target by the year 2030 may not be achievable in Nigeria and globally without increased attention to prevent transmission from the millions of people who have subclinical TB by moving beyond symptom-only ACF methods.^1^ Based on the reality of subclinical TB regarding TB transmission in Nigeria, the KNCV Nigeria has adopted the use of both PDX with AI and WHO 4-symptom screen (W4SS) (i.e., parallel TB screening with any TB symptom and CXR)^3^ for systematic TB screening in Nigeria. In this study, KNCV Nigeria wished to use the data from the project states to provide community TB ACF-based evidence on the magnitude and characteristics of subclinical TB in Nigeria. The study results would guide the understanding of TB disease progression and control guidelines in Nigeria and other TB-high-burden countries.

## STUDY POPULATION, DESIGN AND METHODS

The KNCV Nigeria implements Nigeria’s USAID-funded Tuberculosis Local Organizations Network (TB-LON) Regions 1 and 2 Project. This study retrospectively analysed the data of community ACF TB interventions in six project states where the parallel TB screening with any TB symptom and CXR (with AI) algorithm was used.^3^ The intervention sites include farm camps, mosques, correctional facilities, schools, markets, and churches. The six project states included two from the Southern region of Nigeria (Cross River and Delta) and four from the northern region (Benue, Kano, Katsina, and Nasarawa). All states are high-burden TB states with a median case notification rate (CNR) of 72/100,000 population (range: 49–100/100,100).^11^ The study period was from January 2022 to May 2023.

**FIGURE 2.**
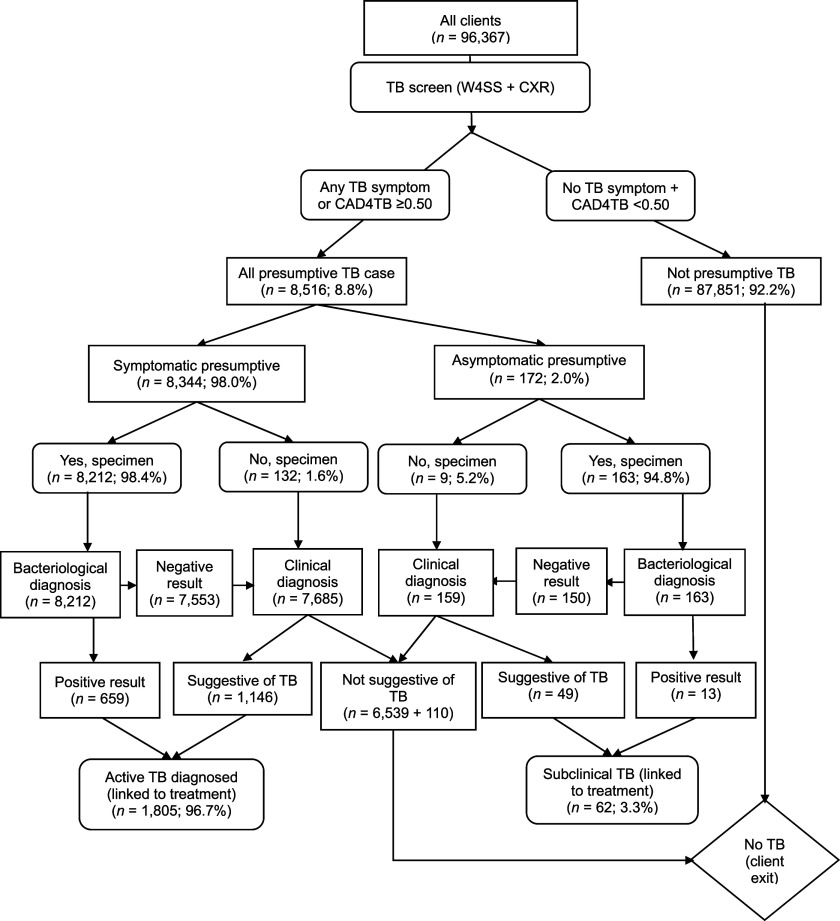
The screening/diagnostic algorithm used for the study’s ACF project. W4SS = WHO four-symptom screen; CXR = chest X-ray; CAD4TB = Computer Aided Detection for TB; ACF = active case-finding.

During the ACF project, each consenting client underwent symptom screening using the WHO-recommended four-symptom screen (W4SS) and CXR screening using a portable digital X-ray (PDX) with AI. The AI is anchored on computer-aided detection for TB (CAD4TB) software associated with the PDX. A client with any primary W4SS symptoms and/or CAD4TB score of ≥0.5 was adjudged as presumptive. All eligible presumptive had TB evaluation using the available bacteriological methods with nucleic-acid amplification test (NAAT) for those who could produce specimens (sputum for adults or stool for children). The available molecular diagnostic tests were the Xpert^®^ MTB/RIF assay (Cepheid, Sunnyvale, CA, USA), Truenat (Molbio Diagnostics, Verna, India), and TB-LAMP (Eiken Chemical Company Ltd, Tokyo, Japan). On the other hand, expert (radiologist) review of chest radiogram was used for clinical TB diagnosis among presumptive who could not produce appropriate specimens and those whose bacteriological tests were negative (i.e., sequential confirmatory test algorithm).^12^ As described in the introduction, subclinical TB is a presumptive that was W4SS screen-negative but positive to either bacteriological TB diagnostic test or clinical TB diagnosis by a radiologist.^4^ The screening/diagnostic algorithm used for this study’s ACF project is illustrated in Figure 2. Details of each presumptive, including the name, age, TB symptoms and TB diagnostic test(s) results, were recorded in the electronic presumptive register of the project state.

The study’s primary outcome measure is the percentage of subclinical TB among all TB cases and the prevalence of subclinical TB among asymptomatic presumptive. The demographic characteristics (sex and age), capacity to produce specimens, history of TB treatment/contact with cough patients, and use of clinical TB diagnosis were compared between subclinical TB and symptomatic TB groups.

Verbal informed consent was received from every client and legal representative of minors involved in the ACF TB interventions used in the study. The study was approved by the National Health Research Ethics Committee of Nigeria (NHREC; Abuja, Nigeria; approval number is NHREC/01/01/2007-04/10/2023.

MS Excel (Microsoft, Seattle, WA, USA) was used to merge, clean, and de-identify relevant data from the electronic presumptive registers of all project states. IBM SPSS v27 (IBM, Armonk, NY, USA) was used for descriptive and inferential data analyses. Continuous data were compared with the independent *t*-test, while categorical data were compared with the Pearson χ^2^ or Fisher’s exact test. A *P*-value of less than 0.05 was adopted as statistically significant.

## RESULTS

Data from 8,516 presumptive (male: 5,172; 60.7%; female: 3,344; 39.3%) across the six project states were adequate for inclusion in the study and were analysed. The median age was 38 years (interquartile range: 24–55), while the modal age group was 25–34 years (*n* = 1,526; 17.9%).

One hundred and seventy-two (2.0%) presumptive had no identifiable TB symptom, i.e., W4SS screen-negative but CXR screen-positive, while the remaining were distributed between W4SS screen-only positive (*n* = 5,646; 66.3%), and W4SS plus CRX positive (*n* = 2,698; 31.7%). As shown in Table 1, the sex distribution of presumptive without TB symptoms was 127 (73.8%) males and 45 (26.2%) females. The 25–34-year group was the most common age group among all clients (18.1%), all presumptive (17.9) and asymptomatic presumptive (24.4%).

**TABLE 1.** Sex and age group distribution of clients, presumptive types and all TB cases.

Category	All presumptive TB	Presumptive without symptoms	TB cases among all presumptives
	(*n* = 8,516)	(*n* = 172)	(*n* = 1,867)
	*n* (%)	*n* (%)	*n* (%)
Sex			
Male	5,172 (60.7)	127 (73.8)	1,345 (72.0)
Female	3,344 (39.3)	45 (26.2)	522 (28.0)
Age group, years			
0–4	80 (0.9)	0 (0.0)	2 (0.1)
5–14	793 (9.3)	7 (4.1)	53 (2.8)
15–24	1,277 (15.0)	31 (18.0)	177 (9.5)
25–34	1,526 (17.9)	42 (24.4)	367 (19.7)
35–44	1,452 (17.1)	36 (20.9)	354 (19.0)
45-54	1,159 (13.6)	20 (11.6)	280 (15.0)
55–64	911 (10.7)	20 (11.6)	232 (12.4)
≥65	1,318 (15.5)	16 (9.3)	402 (21.5)

A total of 1,867 (21.9%) TB cases were diagnosed among all presumptive, which translates to a rate of 21,924 per 100,000 presumptives. Sixty-two (3.3%) TB cases were subclinical, while 1,805 (97.3%) were active TB. The 62 subclinical TB cases were diagnosed from 172 asymptomatic presumptive, giving a subclinical TB prevalence of 36.0%, which was significantly higher than the active TB prevalence among symptomatic presumptive (21.6%, 1,805/8,344; *P* < 0.001, odds ratio [OR] = 2.0, 95% confidence interval [CI] 1.49–2.80). The mean age of subclinical TB cases (42.3 ± 18.2 years) was similar to that of active (symptomatic) TB cases (45.9 ± 19.3 years) (*P* = 0.134).

As shown in Table 2, the sex distribution was similar between the subclinical and active TB groups (*P* = 0.337). The modal age group of the subclinical TB group was 25–34 years, while that of the active TB group was ≥65 years. There were no cases of subclinical TB within the 0–4 years and 5–14 years age groups. Only one of the subclinical TB cases (1.6%) had resistant TB. None of the subclinical TB cases had a history of TB treatment compared to the active TB group, where 268 (14.8%) were recurrent TB cases (*P* < 0.001). Also, there was no history of contact with a cough patient among the subclinical TB cases compared to the 78.1% among the active TB cases (*P* < 0.001).

**TABLE 2.** Distribution of subclinical and symptomatic TB groups by diagnostic test mode and other characteristics.

Category	Sub-category	Subclinical TB group (*n* = 62) *n* (%)	Symptomatic TB group (*n* = 1,805) *n* (%)	*P* value	OR (95%CI)
Sex	Male	48 (77.4)	1,297 (71.9)	0.337	1.3 (0.73–2.46)
	Female	14 (22.6)	508 (28.1)		
Age group, years	0–4	0 (0.0)	2 (0.1)	0.133	—
	5–14	0 (0.0)	53 (2.9)		
	15–24	9 (14.5)	168 (9.3)		
	25–34	17 (27.4)	350 (19.4)		
	35–44	12 (19.4)	342 (18.9)		
	45-54	8 (12.9)	272 (15.1)		
	55–64	9 (14.5)	223 (12.4)		
	≥65	7 (11.3)	395 (21.9)		
History of TB treatment	Yes	0 (0.0)	268 (14.8)	<0.001	—
	No	62 (100.0)	1537 (85.2)		
Contact with cough patient	Yes	0 (0.0)	396 (21.9)	<0.001	—
	No	62 (100.0)	1409 (78.1)		
Ability to produce a specimen	Yes	57 (91.9)	1728 (95.7)	0.151	0.5 (0.20–1.30)
	No	5 (8.1)	77 (4.3)		
Mode of TB diagnosis	CXR	49 (79.0)	1146 (63.5)	0.012	2.2 (1.17–4.03)
	Molecular tests	13 (21.0)	659 (36.5)		
Resistant TB	Yes	1 (1.6)	11 (0.6)	0.334	2.7 (0.34–21.04)
	No	61 (98.4)	1794 (99.4)		

OR = odds ratio; CI = confidence interval; CXR = chest X-ray.

Table 3 shows the distribution of subclinical TB cases by TB diagnostic types. Thirteen (22.8%) TB cases were bacteriologically confirmed with molecular tests out of the 57 subclinical TB that could produce specimens. TB diagnoses for the 44 subclinical TB that tested negative to the molecular tests were clinical, by radiologists’ review of their CXR.

**TABLE 3.** Distribution of subclinical TB cases by TB diagnostic tests.

Specimen production status	Diagnostic test type	Total TB cases tested *n* (%)	Molecular test-positive *n* (%)	Molecular test-negative/TB diagnosis using CXR expert review *n* (%)
Yes (*n* = 57)	Xpert (Cepheid, Sunnyvale, CA, USA)	43 (75.4)	7 (16.3)	36 (81.8)
	Truenat (Molbio, Verna, India)	13 (22.8)	5 (38.5)	8 (18.2)
	TB-LAMP (Eiken Chemical Company Ltd, Tokyo, Japan)	1 (1.8)	1 (100.0)	0 (0.0)
	Sub-total*	57 (100.0)	13 (100.0)	44 (100.0)
No (*n* = 5)	CXR (expert review)	5 (100.0)	—	5 (100.0)
Total^†^		62 (100.0)	—	49 (100.0)

* Subtotal for each column under TB cases that produced specimen.

† Combined total for each column under all TB cases irrespective of specimen production.

CXR = chest X-ray.

The ability to produce specimens for molecular TB testing did not differ between the subclinical and active TB groups (*P* = 0.151). Furthermore, though clinical diagnosis by the expert review of CXR was the predominant method of TB diagnosis for both groups, the proportion in the subclinical TB group (49/62, 79.0%) was significantly higher than the active TB group (1,146/1,805, 63.5%; *P* = 0.012, OR = 2.2, 95% CI 1.17–4.03). Details of the ability to produce a specimen, TB burden, and diagnostic test distribution among the subclinical and active TB groups are shown in Table 2.

## DISCUSSION

This study aimed to determine the magnitude of subclinical TB in Nigeria using data from community-based ACF TB that applied the parallel TB screening algorithm,^3^ followed by TB diagnostic testing of presumptive with the sequential confirmatory test algorithm.^12^ It found that subclinical TB constituted 3.3% of the large number of prevalent TB cases identified. This proportion may appear small, but when translated to absolute numbers, it means a TB case rate of 724/100,000 presumptives, which is enormous. Nevertheless, the percentage of subclinical TB in this study is far less than the median percentage of 49.4% identified among African countries in a review of national TB surveys,^6^ and 18.2% reported in a hospital-based study in Suzhou, China.^5^ Also, the percentage of subclinical TB in this study is less than the 22.9% identified from the report of the first national TB prevalence survey in Nigeria.^13^ The Nigerian TB survey defined symptomatic presumptive as a prolonged cough of at least 2 weeks (or CXR abnormality suggestive of TB), unlike this study that used the W4SS (and CXR with AI); thus, it probably underestimated the active TB proportion which might have contributed to the observed wide disparity in the percentage of subclinical TB between the survey and the current study. A study among household contacts in South Africa showed a preponderance of subclinical TB among women when compared to men;^14^ but, in this study, subclinical TB did not seem to have any peculiar demographic characteristics – the sex and age distributions were similar to the active TB (Table 2). A few active TB cases were identified in the 5-14 age group, but it’s unclear why no subclinical TB was found among child presumptive, despite subclinical TB being well-documented in children – for instance, a subclinical TB proportion of up to 31% was reported from Switzerland, and most of the cases were less than 5 years.^15^

It has been established that undiagnosed and untreated TB in the community fuels TB transmission globally.^16^ Notably, a part of this undiagnosed TB is the subclinical TB missed during symptom-only TB screening. As shown in this study, as high as 36% of asymptomatic presumptive were diagnosed with TB, which suggests that all of them would have been missed if the TB screening method was symptom-only. This finding highlights the suboptimal TB case detection capacity of symptom-only TB screening and underscores the need to upgrade such screening methods to algorithms that include CXR for asymptomatic individuals at the least.^3^ The need for a more effective TB screening algorithm that covers asymptomatic individuals is strengthened by this study’s finding that the odds of being diagnosed with TB among asymptomatic presumptive was more than twice that of symptomatic presumptive. It is known that the parallel screening with any TB symptom and CXR screening algorithm is associated with the highest TB cases (about 85%) in TB endemic areas such as Nigeria.^3^ Given the high TB case yield in this study, it is likely that using CXR with AI in TB-endemic settings increases the detection of TB cases. As shown in Figure 1, subclinical TB cases are infectious and can transmit the disease in the community; thus, they constitute a serious public health concern. This concern is supported by a recent review of data from 14 countries across Asia and Africa, which estimated that subclinical TB contributes 68% of global TB transmission relative to active TB.^17^

Outside highlighting that subclinical TB has a similar capacity with active TB to produce sputum, this study also showed that a majority of the subclinical TB were diagnosed clinically by radiologists’ review of their CXR. This observation is policy-relevant because it shows the importance of having a ready pool of trained radiologists for expert review of relevant CXR in every setting where the TB screening algorithm involves CXR (with or without AI).

Furthermore, it is noteworthy that molecular diagnostic tests were negative in up to 77% of subclinical TB that submitted specimens in this study (Table 3). While this finding supports the importance of clinical TB diagnosis, which had higher odds among subclinical TB compared to active TB (Table 2), it reinforces the knowledge that molecular diagnostic tests can produce false negative results among patients with pulmonary tuberculosis; a recent study of 2636 presumptive from two states of Nigeria found Xpert-negative/culture-positive results in 20 TB cases.^18^

To the best of our knowledge, this study is the first effort in Nigeria focused on quantifying the magnitude of subclinical TB among all TB cases in community-based ACF TB projects, and the findings will hopefully stimulate more studies on the subject. As expected, the retrospective nature of the study limited the scope. For instance, it has been established that living with HIV is associated with TB disease.^19^ However, the study could not assess the association between HIV-positive status and subclinical TB because the information was not available.

In conclusion, the TB burden of 21,924/100,000 presumptives was identified in this study, and subclinical TB contributed 3.3% of this enormous burden. Notably, the subclinical TB cases would have been missed if the symptom-only TB screening had been employed for the ACF TB project. We recommend that the NTLCP revise the traditional binary TB classification (latent and active) in the national guidelines and discourage symptom-only TB ACF in Nigeria.
